# High-Energy Extracorporeal Shock Wave for Early Stage Osteonecrosis of the Femoral Head: A Single-Center Case Series

**DOI:** 10.1155/2015/468090

**Published:** 2015-11-02

**Authors:** Fuqiang Gao, Wei Sun, Zirong Li, Wanshou Guo, Weiguo Wang, Liming Cheng, Bailiang Wang

**Affiliations:** Centre for Osteonecrosis and Joint-Preserving & Reconstruction, Department of Orthopedic Surgery, Beijing Key Laboratory of Arthritic and Rheumatic Diseases, China-Japan Friendship Hospital, National Health and Family Planning Commission of the People's Republic of China, Beijing 100029, China

## Abstract

Our retrospective study assessed the effects of treatment of early stage ONFH with extracorporeal shock wave therapy. 335 patients (528 hips) were treated with shockwave therapy in our institution. Each patient underwent two sessions. The hips were divided into two groups according to whether the lateral pillar of the femoral head (LPFH) was preserved: LPFH and non-LPFH groups. Patients were followed up at 3, 6, and 12 months after the treatment. Most of the patients (83.9% hips) demonstrated pain reduction and improved mobility of the treated joint (visual analogue scale score, *P* = 0.00006; Harris hip score, *P* = 0.00091). During the follow-up period, 16 hips failed following femoral head collapse and required hip arthroplasty (2 hips in LPFH group and 14 hips in non-LPFH group). The lesion size decreased after ESWT. However, the differences were statistically not significant (LPFH group, *P* = 0.091; non-LPFH group, *P* = 0.087). A significant reduction in bone marrow edema was observed after treatment (LPFH group, *P* = 0.007; non-LPFH group, *P* = 0.016). High-energy extracorporeal shock wave therapy resulted in considerable improvement in early stage ONFH, which can effectively relieve pain and improve the function of the hip.

## 1. Introduction

The osteonecrosis of the femoral head (ONFH) is common in young adults, and it is the leading cause of hip joint replacements in many Asian countries including China [1]. Although the disease remains well-known, it is not fully understood because of the difficulty in early diagnosis, miscellaneous etiologies, unclear pathogenesis, and undetermined successful treatment [2]. Treatment of this disease remains controversial [3, 4]. Various efforts have been made in an attempt to enhance the healing of osseous defects in the femoral head before collapse occurs. The treatment approach is determined based on early diagnosis and preservation of the affected hip [1, 5].

Treatment of ONFH is dependent on the stage, size, and location of the lesion. The ONFH adults with Stages I to III present an overall therapeutic challenge [6]. Conservative treatments such as NSAID, physical therapy, and protected weight bearing [3, 5] are generally unsuccessful, and a variety of surgery procedures varying according to the stage of the disease on image studies are indicated in symptomatic hips [3–8]. For early stage ONFH, hip-preserving procedures including core decompression, vascularized or nonvascularized bone graft, muscle pedicle graft, and derotational osteotomy are recommended [2, 4, 5, 7]. However, most studies reported that the results of these procedures varied considerably, with less satisfactory outcomes [2–7]. For many patients with advanced disease, total hip arthroplasty (THA) was performed [3, 4, 8]. However, a few complications of THA including thigh pain, polyethylene wear, osteolysis, and component loosening have been reported in young active patients [4, 8]. Therefore, there is an unmet need for an effective and noninvasive alternative method for treating ONFH.

Extracorporeal shockwave therapy (ESWT) is an invasive therapeutic approach that has shown efficacy in the treatment of certain orthopedic conditions such as nonunion of long-bone fracture [9–13]. More recently, the results of shock wave therapy in ONFH have been encouraging. It appeared to be more effective than core decompression and nonvascularized bone grafting for early ONFH [9, 10]. ESWT provided beneficial effects for hips affected by ONFH in patients. ESWT may have the potential to shorten the progression of the disease and to postpone the need for hip arthroplasty in young patients [13]. However, the mechanism by which shock wave treatment results in clinical improvement remains unclear. Some have postulated that shock wave therapy provokes a painful level of stimulation and relieves pain by hyperstimulation analgesia, while others have speculated that shock wave therapy produces microfractures and activates cells to express genes for osteogenesis which in turn causes new bone formation [9, 14].

Previous studies have shown favorable results on the efficacy of ESWT in the treatment of early ONFH. However, the sample sizes were relatively small and lack conviction. The objective of the retrospective study was to show the effectiveness of high-energy shock wave therapy in the treatment of early stage necrosis of the femoral head by assessing clinical and magnetic resonance imaging (MRI) results in a large case study.

## 2. Materials and Methods

The study was approved by the Institutional Review Board on Human Studies of the Ethical Committee of our hospital.

### 2.1. Clinical Data

ESWT considered for the included patients at our institution was not only based on their clinical symptoms. We included patients with Stage I, II, and III ONFH according to the ARCO classification [15], mentioned in the literatures [4, 13]. We excluded patients with acute infection, patients on immunosuppressant drugs for malignancy (except corticosteroids), patients with coagulation disorders, patients with cardiac arrhythmia requiring a pacemaker, patients who were pregnant, patients with skeletal immaturity, and patients with poor compliance [16]. Between January 24, 2012, and December 1, 2013, 335 patients (528 hips) with early ONFH were recruited in the study (Table 1).

**Figure 1:**
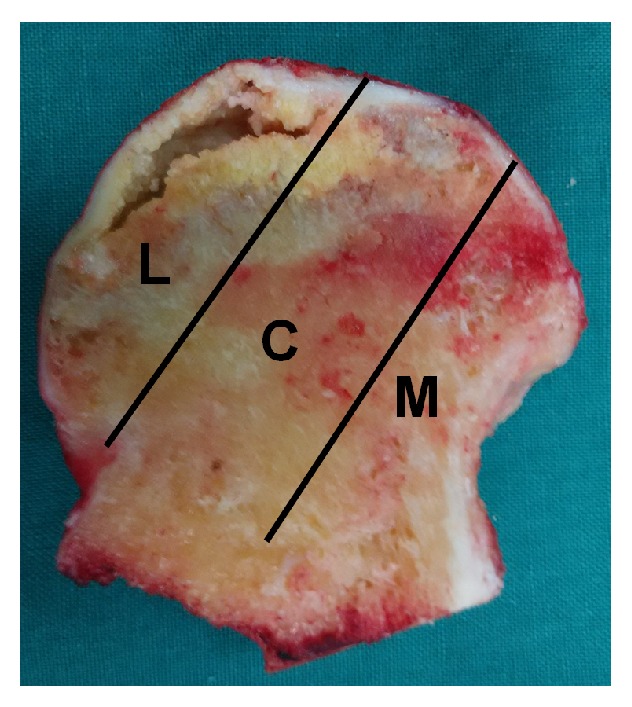
Image of coronal section of the femoral head showing three pillars of the femoral head: lateral (30%), central (40%), and medial (30%) [19].

**Figure 2:**
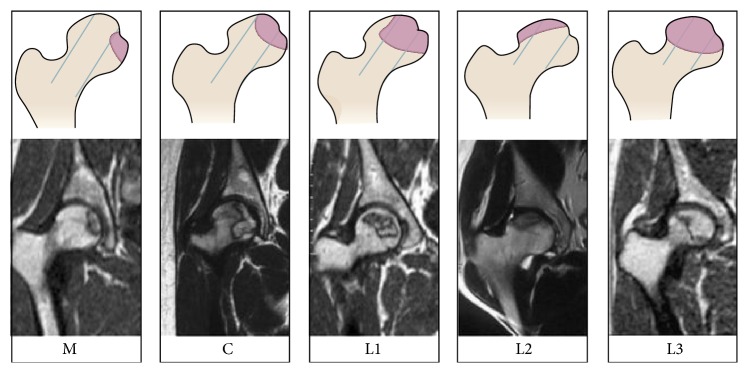
Schematic diagram and MRI of China-Japan Friendship Hospital (CJFH) classification for osteonecrosis of the femoral head based on three pillars [20]. Type M: the necrosis involved the medial pillar. Type C: the necrosis involved both medial and central pillars. Type L1: the necrosis involved the three pillars but the partial lateral pillar was preserved. Type L2: the necrosis involved whole lateral pillar and partial central pillar. Type L3: the necrosis involved the three pillars including the cortical bone and marrow.

Preoperative clinical evaluations included a complete history and physical examination, laboratory tests including CBC, platelet count, PT, PTT, chemistry profiles, BUN and creatine, EKG and chest X-rays, radiographs, and MRI of the affected hip or hips. All hips were symptomatic on evaluation. The underlying diagnosis was confirmed by X-rays and MRI. All subjects received an MRI evaluation according to China-Japan Friendship Hospital (CJFH) classification [19, 20] (Figures 1 and 2) for osteonecrosis of the femoral head based on three pillars (Figure 1). The hips were divided into two groups according to whether the lateral pillar of the femoral head (LPFH) was preserved or not [21] (Table 2). The LPFH group consisted of 376 hips with the preservation of the lateral pillar of the femoral head (including CJFH Types M, C, and L1). The non-LPFH group consisted of 152 hips without the preservation of the lateral pillar of the femoral head (including CJFH Types L2 and L3).

### 2.2. Shock Wave Treatment

**Figure 3:**
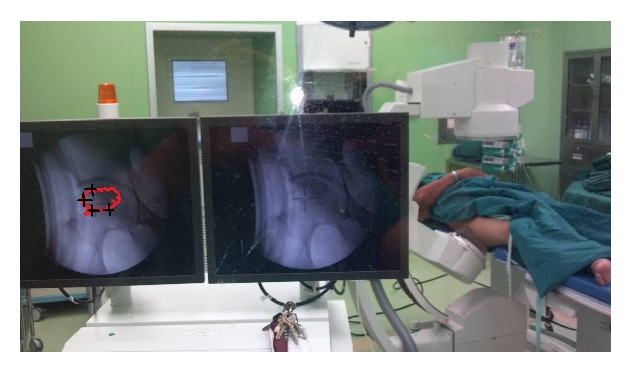
Image showing shockwave treatment of ONFH. Shock waves are applied under X-ray guidance. Four to six treatment points are located on the hardened layer around the necrosis lesion.

Although small areas of necrosis may remain asymptomatic and resolve spontaneously, most of the clinically diagnosed cases involving the hip progress without treatment to collapse and eventual arthroplasty [2–4]. Our goal therefore is to prevent femoral head collapse and to preserve rather than to replace the joint. All patients were required to sign a consent for shock wave treatment in the study. All patients were recruited to the study after their treatment. The shock wave treatment was applied using an Electromagnetic Shock Wave Emitter (Dornier Compact DELTA II, Munich, Germany) (Figure 3), with a penetration depth between 0 and 150 mm and a focus diameter of 4 mm. The shockwave tube generating a shock wave was directed to the skin surface near the greater trochanter of femur. The shock waves were focused around (on the margins of) the femoral head under radiographic guidance. The functional zone between avascular and normal bones of the femoral head was delineated by C-arm imaging (Figure 3). Four to six points were located on the hardened layer around the necrotic lesion [4, 6, 12, 13, 16] (Figure 3). The treatment area was prepared with a coupling gel to minimize the loss of shock wave energy at the interface between the head of the device and the skin.

All ESWT procedures were performed once without general or regional anesthesia by experienced physicians with the patient in supine position on the operation table. ESWT orthopedic settings were prepared and used according to the methods described by Wang et al. [4, 12, 13, 16] as follows: number of levels, 3-4; each spot received a dose of 500–1000 pulses at an energy flux density of >0.44 mJ/mm^2^ (level 3) and 3000–4000 impulses at a frequency of 2-3 Hz. Each patient underwent two therapy sessions (the time interval between successive procedures was one week). After ESWT treatment, patients were instructed to walk on crutches with partial weight bearing on the affected leg for 4–6 weeks. Alendronate sodium tablets (70 mg p.o. q.w. for 12 months) were administered to each patient. Nonnarcotic analgesics such as celecoxib were prescribed for pain. Patients were followed up at the outpatient department at 3, 6, and 12 months after the second procedure. An assessment of pain intensity (visual analogue scale, VAS) and hip function (Harris hip score, HHS) was carried out before and after the therapy. Radiographic assessment was performed using plain radiographs and MRI. Radiographs were used to assess the size and location of the lesion, congruency of the femoral head, presence of a crescent sign, and degenerative changes of the hip joint. We used MRI findings to evaluate the changes in lesion size, the congruency of the articular surface, and bone marrow edema. No supplemental calcium was given to the patients in this study. Radiographs and MRI of the affected hip were performed preoperatively, at 6 and 12 months, and once a year thereafter. The primary endpoint of the study was the need for total hip arthroplasty (THA) during the course of treatment. Secondary endpoints included improvement in pain and function of the affected hip and changes on X-ray and MR images, including the size of the lesion and bone marrow edema.

### 2.3. Statistical Analysis

We compared pain and Harris hip scores before and after the shock wave treatment using paired* t*-tests. The overall clinical outcomes and the changes in lesion size were compared statistically using a chi-square test for statistical significance using a 95% confidence interval (*P* < 0.05). All data analyses were performed using SPSS version 16.0.0 software (SPSS; Chicago, IL). All results are expressed as mean ± standard deviation (SD).

## 3. Results

### 3.1. Clinical Results

In the current study, 335 patients (528 hips) were treated with shockwave therapy in our institution, between January 24, 2012, and December 1, 2013 (Tables 1 and 2). The study population consisted of 106 women and 229 men with a mean age of 43.7 ± 13.7 years. There was a significant improvement in pain scores (VAS) and Harris hip scores after the treatment. Most of the patients (83.9% hips) showed pain reduction and improved mobility of the treated joint. The mean VAS score for both groups decreased from 6.8 ± 3.7 to 1.0 ± 2.1 (*P* = 0.00006). The mean Harris hip score for both groups increased from 69.4 ± 14.7 to 90.9 ± 11.4 (*P* = 0.00091). Most patients described the daily life function as significantly improved, but the Harris score after the removal of the pain score increased slightly from 36.1 ± 7.9 before treatment to 40.6 ± 13.5 after treatment, and the difference was not statistically significant (*P* > 0.05), which indicated that its function improvement was mainly due to the reduction of pain.

Table 3 summarizes the results of clinical assessment before and after the treatment. At the last follow-up time (a minimum of 12 months), ESWT might have the potential to curtail the progression of the disease and to delay the need for THA. 86.2% (324/376) of the hips in the LPFH group and 78.3% (119/152) in the non-LPFH group showed improvement. However, for some CJFH Type L2 and L3 osteonecrosis, the treatment outcomes were poor or inadequate. 14 hips in the non-LPFH group failed following femoral head collapse and required hip arthroplasty, but only 2 hips in the LPFH group failed.

### 3.2. Radiological Results

**Figure 4:**
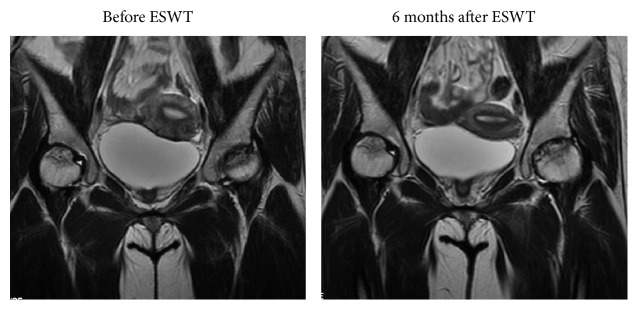
MR images of the bilateral hips in a 31-year-old female patient with glucocorticoid-induced osteonecrosis of the femoral head showed regression of the lesion 6 months after ESWT, and the hips were pain-free for daily activities.

**Figure 5:**
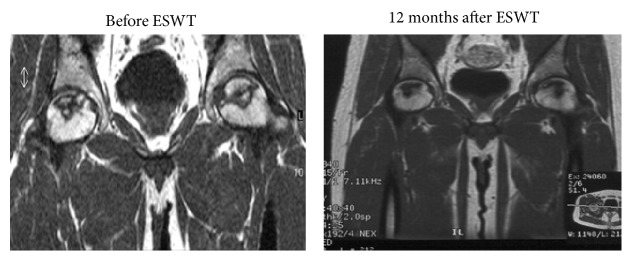
Radiographs of the bilateral hips before and after treatment in a 21-year-old male patient with osteonecrosis of the femoral head showing a trend of decrease in the size of the lesion after ESWT and no changes in the stages of the disease and no further collapse of the femoral heads.

**Figure 6:**
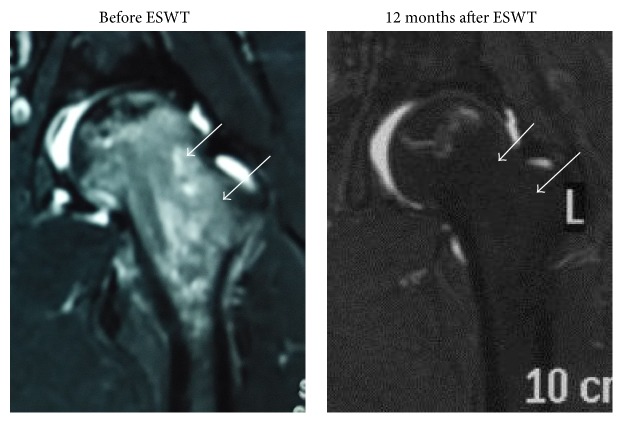
MRIs of the left hip before and after treatment showing resolution of bone marrow edema and no further collapse of the femoral heads (Arrow).

Table 4 summarizes the results of radiographic and MRI evaluations before and after treatment. There was a trend of decrease in the size of the lesion after ESWT (Figures 4 and 5). However, the differences were not statistically significant (*P* > 0.05). It was easier to visualize the bone marrow edema in MRI of ONFH in non-LPFH group (205/376 hips, 54.5%) than in LPFH group (130/152 hips, 85.5%). A significant reduction in bone marrow edema was observed after treatment (*P* < 0.05) (Figure 6). The reduction in bone marrow edema correlated with clinical improvement in pain and function of the hip [18]. The imaging studies showed stable images of the hip, including the obvious osteogenesis signs of the femoral head, absence of progress in osteonecrosis staging, and significantly improved concomitant bone marrow edema (Table 4).

### 3.3. Complications

**Figure 7:**
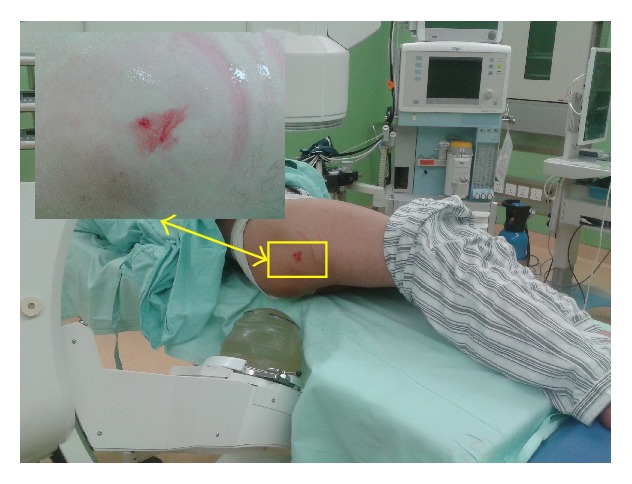
Mild local swelling and erythema at the treatment site in the greater trochanter area after ESWT.

There were no systemic or neurovascular complications. Mild local swelling and erythema at the treatment site in the greater trochanter area (Figure 7) were noted in 171 of 528 hips (32.4%) in all patients. Observation caused the edema to resolve within a few days. There were no device-related problems or complications.

## 4. Discussion

The natural history of ONFH usually results in collapse of the femoral head and deterioration with degenerative changes of the hip, and surgery becomes inevitable [4, 22]. The pathophysiology of this disease is vague for most cases with speculation of vascular injury and changes in cell biology [23]. Currently, there is no gold standard for absolutely effective treatment of ONFH. The conservative treatments are generally not successful, and the choice of surgery varies depending on the stage of the disease [3]. Core decompression with or without bone grafting is the most common procedure performed for symptomatic hips affected by ONFH [7] via relieving the intraosseous pressure of the femoral head and promoting the remodeling and regeneration of the femoral head [4, 24]. However, the results of core decompression varied widely ranging from 29% to 84%, and most results were unsatisfactory in the reported literature [16].

While core decompression is the most commonly employed method for femoral head preservation after ONFH, several recent articles have reported efficacy with ESWT [10, 11, 16, 22]. ESWT was recently utilized in the treatment of early ONFH. The response to ESWT was shown to be effective in early ONFH [4, 13, 25–27] with 79% clinical improvement and 39% regression of the lesion on MRI [16]. The EWST appeared to be more effective than core decompression and nonvascularized fibular grafting in patients with early stage osteonecrosis of the femoral head although the mechanism by which the shock wave treatment results in clinical improvement remains unknown [16]. Significant improvements in pain and function were noted at each time interval favoring the ESWT. There was a trend of decrease in the size of the lesion in the ESWT group [4, 16]. It appears that the application of shockwave resulted in regenerative effects in hips with ONFH, which is consistent with our study results. The application of ESWT is found to be effective in the retardation or prevention of collapse of the femoral head in early ONFH including corticosteroid-induced ONFH in patients with systemic lupus erythematosus [13]. Koo et al. [28] reported a correlation with hip pain and bone marrow edema in hips affected by ONFH. In our study, the findings confirmed this and showed significant improvement in pain and function of affected hips and reduction in bone marrow edema on MRIs after shockwave treatment. The shockwave treatment altered the natural course of hips affected by ONFH [22]. Our findings provide further evidence that high-energy extracorporeal shock wave treatment may be an effective noninvasive method for treatment of ONFH.

Despite good clinical results, the exact mechanism of shockwave in ONFH remains poorly understood. Applied to bone, shock waves selected with the appropriate energy and number of pulses can stimulate osteogenesis and angiogenesis [9, 11, 12]. Recent studies unveiled that ESWT induces neovascularization, upregulates angiogenesis and osteogenesis-related growth factors including eNOS (endothelial nitric oxide synthase), VEGF (vessel endothelial growth factor), PCNA (proliferating cell nuclear antigen), and BMP-2 (bone morphogenetic protein 2), and promotes cell proliferation and differentiation leading to tissue regeneration [9, 29]. Nitric oxide and VEGF are important mediators of angiogenesis [12, 29]. In experiment in rabbits, ESWT was shown to increase BMP-2 protein and mRNA and upregulation of VEGF expression in necrotic subchondral bone of the femoral head, which may mainly induce the ingrowth of neovascularization associated with increased expressions of angiogenic growth factors and improvement in blood supply to the femoral head that in return promotes bone remodeling and regeneration in hips with ONFH [9, 11]. Furthermore, ESWT can promote bone marrow stromal cell (BMSC) differentiation toward osteoprogenitors associated with induction of TGF-p1 and induces membrane hyperpolarization and Ras activation to act as an early signal for osteogenesis in human bone marrow stromal cells (BMSCs) [12, 30]. ESWT significantly enhanced the angiogenic and osteogenic effects of BMSCs mediated through the nitric oxide (NO) pathway in hips with osteonecrosis [31]. Shock wave therapy promotes early release of angiogenic factors and subsequently induces cell proliferations and ingrowth of neovessels that in turn may stimulate the stromal cell growth and differentiation and promote bone healing [12].

As mentioned, ESWT-treated femoral heads showed significant increases in angiogenesis with new vessel formation and cell proliferation, bone remodeling, and regeneration. However, loss of mechanical integrity may predispose them to subchondral fracture and failure of the disease if the increased vascularity and bone remodeling do not necessarily assure bone resorption [4]. Therefore, ESWT is best applied in hips with early stage ONFH before the development of the crescent sign. These findings are in concert with our findings with clinical observation and the analysis of therapeutic outcome. We hold the opinion that the collapse of ONFH is closely related to whether the necrotic foci occupy the lateral pillar of the femoral head and the degree of involvement [21]. Our previous studies showed that whether ONFH progressed to collapse is determined by preservation of the lateral pillar. When the lateral pillar is preserved, the collapse rate is low and the femoral head maintains the spherical shape for a long time. Most femoral heads will collapse in a short time when the lateral pillar is involved completely. This study confirmed that the necrotic foci, the lateral pillar of the femoral head, and the degree of involvement directly affect the treatment effect of the shock wave. It appeared that shockwave treatment altered the progress course of hips affected by ONFH when the lateral pillars were completely involved. The lateral pillar is the keystone for maintaining the sphere of the femoral head and its preservation. The results obtained thus far with high-energy shock wave therapy in these patients suggest that this noninvasive and moderately priced method may offer an alternative to invasive treatment modalities for femoral head necrosis, especially when the lateral pillar is preserved.

There are some limitations in this study. So far, the mechanisms and indications of ESWT have not been very clear. The indications are mainly based on the supported literatures and our previous clinical observation. This study is limited by virtue of the retrospective analysis. There was no randomized and blinded control group with conservative treatment in this study. However, we believe that the methods and results in larger patient population in this study do not affect the overall outcomes. Furthermore, patients with different primary diseases such as SLE can pose many medical variables including administration of corticosteroids to control the symptoms that may directly or indirectly affect the results of treatment. The treatment with corticosteroids has relatively serious side effects on bone tissue. But the differences in the use of corticosteroids between both groups were statistically not significant (LPFH group, 51.1% (192/376 hips); non-LPFH group, 55.9% (85/152 hips); *χ* ^2^ = 0.090, *P* = 0.765). It may not affect the final outcomes. The follow-up time of this study is relatively short. The results during this 1-year study may not necessarily represent the long-term results. Long-term results are needed to confirm the effectiveness of ESWT for hip necrosis.

In conclusion, extracorporeal shockwave treatment provided beneficial effects for hips affected by early ONFH in the short term. This novel treatment modality might have the potential to curtail the progression of the disease and to delay the need for THA. However, for some CJFH Type L2 and L3 osteonecrosis, the treatment outcomes were poor or inadequate. Long-term studies are warranted to confirm the long-term effects of ESWT in hip necrosis.

## Figures and Tables

**Table 1:** Patient demographic characteristics.

Demographics	Values
Gender (male : female)	229 : 106
Age	43.7 ± 13.7
Cause	
Steroid	178
Alcohol	81
Idiopathic	67
Other	9
Underlying disease	
Rheumatic and autoimmune disease	81
Hematopathy	64
Dermatosis	15
Transplantation	8
Severe trauma	7
Hormone abuse	2
Other	1
Duration of symptoms (M)	5.7 ± 8.9
ARCO stage lesions (hips)	
Stage I	137
Stage II	246
Stage III	145
CJFH classification (hips)	
Type M	81
Type C	184
Type L1	111
Type L2	57
Type L3	95
Length of follow-up (M)	14.9 ± 9.7

Note: M: month; CJFH: China-Japan Friendship Hospital; ARCO: the Association Research Circulation Osseous.

**Table 2:** The clinical characteristics of the affected hips of all ONFH patients in this study.

	LPFH group	Non-LPFH group
*n* (hips)	376	152
Duration of symptoms, months	6.5 ± 7.1	5.2 ± 9.4
CJFH classification (hips)	M,81; C,184; L1,111	L2,57; L3,95
ARCO stage lesions (hips)		
Stage I	112	25
Stage II	208	38
Stage III	56	89
Medical history		
Steroid intake	192	85
Alcoholic abuse	71	25
Negative	113	42
Length of follow-up (months)	14.5 ± 8.3	15.2 ± 7.8

Note: CJFH: China-Japan Friendship Hospital; ARCO: the Association Research Circulation Osseous.

**Table 3:** The clinical outcome before and after shock wave treatment.

	Before ESWT	After ESWT	*P* value (II)
VAS			
LPFH group (*n* = 376 hips)	4.5 ± 2.4	0.9 ± 1.3	<0.001
Non-LPFH group (*n* = 152 hips)	7.8 ± 3.6	1.2 ± 1.4	<0.001
*P* value (I)	0.006	0.523	
HHS			
LPFH group (*n* = 376 hips)	83.2 ± 11.3	93.8 ± 10.4	0.021
Non-LPFH group (*n* = 152 hips)	62.9 ± 12.8	88.9 ± 13.5	<0.001
*P* value (I)	0.012	0.218	

	LPFH group (*n* = 376 hips)	non-LPFH group (*n* = 152 hips)	

Clinical outcome^a^			
Improved	86.2% (324/376)	78.3% (119/152)	0.037
Unchanged	13.3% (50/376)	12.5% (19/152)	0.109
Worsened	0.5% (2/376)	9.2% (14/152)	<0.001
*P* value (III)	<0.001		

Note: ESWT: extracorporeal shockwave therapy; VAS: visual analogue scale; HHS: Harris hip score.

*P* value (I): comparison of data between LPFH group and non-LPFH group for pain score and Harris hip score.

*P* value (II): comparison of data before and after ESWT within the same group.

*P* value (III): comparison of data between LPFH group and non-LPFH group for clinical outcome.

^a^Clinical outcome [17]: “improved” was defined when there were significant improvements in pain and function of the affected hip after treatment; “unchanged” was defined when there were very little or no changes after treatment; “worsened” was defined when more pain and less function were noted after treatment.

**Table 4:** Changes on radiograph and MR image before and after treatment.

	LPFH group (*n* = 376 hips)			Non-LPFH group (*n* = 152 hips)		
	Before ESWT	After ESWT^**∗**^	*P* value	Before ESWT	After ESWT^**∗**^	*P* value
ON lesion (%)^a^	23.57 ± 8.91	17.92 ± 8.24	0.091	37.62 ± 9.58	29.78 ± 9.32	0.087
Bone marrow edema^b^ (hips)						
Grade 0	171	226	0.007	22	83	0.016
Grade 1	114	86		41	39	
Grade 2	47	53		67	27	
Grade 3	39	11		13	3	
Grade 4	5	0		9	0	

Note: ^**∗**^the last follow-up time. ON: osteonecrosis; ESWT: extracorporeal shockwave therapy.

^a^The ON lesion (%) represents the percentage of the lesion over the total femoral head surface and is shown in mean ± SD (range).

^b^Bone marrow edema was graded 0 for no bone marrow edema, 1 for perinecrotic bone marrow edema, 2 for bone marrow edema extended into femoral head, 3 for bone marrow edema extended into neck of femur, and 4 for bone marrow edema extended into intertrochanteric region [18].

## References

[1] Sun W., Li Z., Shi Z. (2014). Relationship between post-SARS osteonecrosis and PAI-1 4G/5G gene polymorphisms. *European Journal of Orthopaedic Surgery and Traumatology*.

[2] Lee M. S., Hsieh P.-H., Shih C.-H., Wang C.-J. (2010). Non-traumatic osteonecrosis of the femoral head—from clinical to bench. *Chang Gung Medical Journal*.

[3] Mont M. A., Jones L. C., Hungerford D. S. (2006). Current concepts review—nontraumatic osteonecrosis of the femoral head: ten years later. *The Journal of Bone & Joint Surgery—American Volume*.

[4] Wang C.-J., Wang F.-S., Ko J.-Y. (2008). Extracorporeal shockwave therapy shows regeneration in hip necrosis. *Rheumatology*.

[5] Kim S.-Y., Kim Y.-G., Kim P.-T., Ihn J.-C., Cho B.-C., Koo K.-H. (2005). Vascularized compared with nonvascularized fibular grafts for large osteonecrotic lesions of the femoral head. *The Journal of Bone & Joint Surgery—American Volume*.

[6] Ludwig J., Lauber S., Lauber H.-J., Dreisilker U., Raedel R., Hotzinger H. (2001). High-energy shock wave treatment of femoral head necrosis in adults. *Clinical Orthopaedics and Related Research*.

[7] Iorio R., Healy W. L., Abramowitz A. J., Pfeifer B. A. (1998). Clinical outcome and survivorship analysis of core decompression for early osteonecrosis of the femoral head. *Journal of Arthroplasty*.

[8] Dudkiewicz I., Covo A., Salai M., Israeli A., Amit Y., Chechik A. (2004). Total hip arthroplasty after avascular necrosis of the femoral head: does etiology affect the results?. *Archives of Orthopaedic and Trauma Surgery*.

[9] Ma H.-Z., Zeng B.-F., Li X.-L. (2007). Upregulation of VEGF in subchondral bone of necrotic femoral heads in rabbits with use of extracorporeal shock waves. *Calcified Tissue International*.

[10] Mont M. A., Jones L. C., Seyler T. M., Marulanda G. A., Saleh K. J., Delanois R. E. (2007). New treatment approaches for osteonecrosis of the femoral head: an overview. *Instructional Course Lectures*.

[11] Ma H.-Z., Zeng B.-F., Li X.-L., Chai Y.-M. (2008). Temporal and spatial expression of BMP-2 in sub-chondral bone of necrotic femoral heads in rabbits by use of extracorporeal shock waves. *Acta Orthopaedica*.

[12] Wang C.-J., Wang F.-S., Yang K. D. (2003). Shock wave therapy induces neovascularization at the tendon-bone junction. A study in rabbits. *Journal of Orthopaedic Research*.

[13] Lin P.-C., Wang C.-J., Yang K. D., Wang F.-S., Ko J.-Y., Huang C.-C. (2006). Extracorporeal shockwave treatment of osteonecrosis of the femoral head in systemic lupus erythematosis. *Journal of Arthroplasty*.

[14] Takahashi K., Yamazaki M., Saisu T. (2004). Gene expression for extracellular matrix proteins in shockwave-induced osteogenesis in rats. *Calcified Tissue International*.

[15] Gardeniers J. W. M. (1993). ARCO (Association Research Circulation Osseous) International classification of osteonecrosis. ARCO Committee on Terminology and Staging. Report on the committee meeting at Santiago de Compostella. *ARCO Newsletter*.

[16] Wang C.-J., Wang F.-S., Huang C.-C., Yang K. D., Weng L.-H., Huang H.-Y. (2005). Treatment for osteonecrosis of the femoral head: comparison of extracorporeal shock waves with core decompression and bone-grafting. *The Journal of Bone & Joint Surgery—American Volume*.

[17] van der Waal J. M., Terwee C. B., van der Windt D. A. W. M., Bouter L. M., Dekker J. (2005). The impact of non-traumatic hip and knee disorders on health-related quality of life as measured with the SF-36 or SF-12. A systematic review. *Quality of Life Research*.

[18] Chen J.-M., Hsu S.-L., Wong T., Chou W.-Y., Wang C.-J., Wang F.-S. (2009). Functional outcomes of bilateral hip necrosis: total hip arthroplasty versus extracorporeal shockwave. *Archives of Orthopaedic and Trauma Surgery*.

[19] Herring J. A., Kim H. T., Browne R. (2004). Legg-Calvé-Perthes disease. Part I: classification of radiographs with use of the modified lateral pillar and stulberg classifications. *The Journal of Bone & Joint Surgery—American Volume*.

[20] Li Z.-R., Liu Z.-H., Sun W. (2012). The classification of osteonecrosis of the femoral head based on the three pillars structure: China-Japan Friendship Hospital (CJFH) classification. *Chinese Journal of Orthopaedics*.

[21] Sun W., Li Z.-R., Wang B.-L., Liu B.-L., Zhang Q.-D., Guo W.-S. (2014). Relationship between preservation of the lateral pillar and collapse of the femoral head in patients with osteonecrosis. *Orthopedics*.

[22] Wang C.-J. (2012). Extracorporeal shockwave therapy in musculoskeletal disorders. *Journal of Orthopaedic Surgery and Research*.

[23] Ohzono K., Takaoka K., Saito S., Saito M., Matsui M., Ono K. (1992). Intraosseous arterial architecture in nontraumatic avascular necrosis of the femoral head: microangiographic and histologic study. *Clinical Orthopaedics and Related Research*.

[24] Leung P. C. (1996). Femoral head reconstruction and revascularization: treatment for ischemic necrosis. *Clinical Orthopaedics and Related Research*.

[25] Wang C.-J., Wang F.-S., Yang K. D. (2008). Treatment of osteonecrosis of the hip: comparison of extracorporeal shockwave with shockwave and alendronate. *Archives of Orthopaedic and Trauma Surgery*.

[26] Hsu S.-L., Wang C.-J., Lee M. S.-S., Chan Y.-S., Huang C.-C., Yang K. D. (2010). Cocktail therapy for femoral head necrosis of the hip. *Archives of Orthopaedic and Trauma Surgery*.

[27] Wang C.-J., Ko J. Y., Chan Y. S. (2009). Extracorporeal shockwave for hip necrosis in systemic lupus erythematosus. *Lupus*.

[28] Koo K.-H., Ahn I.-O., Kim R. (1999). Bone marrow edema and associated pain in early stage osteonecrosis of the femoral head: prospective study with serial MR images. *Radiology*.

[29] Babaei S., Stewart D. J. (2002). Overexpression of endothelial NO synthase induces angiogenesis in a co-culture model. *Cardiovascular Research*.

[30] Wang F.-S., Wang C.-J., Huang H.-J., Chung H., Chen R.-F., Yang K. D. (2001). Physical shock wave mediates membrane hyperpolarization and Ras activation for osteogenesis in human bone marrow stromal cells. *Biochemical and Biophysical Research Communications*.

[31] Yin T.-C., Wang C.-J., Yang K. D., Wang F.-S., Sun Y.-C. (2011). Shockwaves enhance the osteogenetic gene expression in marrow stromal cells from hips with osteonecrosis. *Chang Gung Medical Journal*.

